# Faience Waste for the Production of Wall Products

**DOI:** 10.3390/ma14216677

**Published:** 2021-11-05

**Authors:** Kirill Petropavlovskii, Tatiana Novichenkova, Victoria Petropavlovskaya, Mikhail Sulman, Roman Fediuk, Mugahed Amran

**Affiliations:** 1 Moscow State University of Civil Engineering (MGSU), 129337 Moscow, Russia; kspetropavlovsky@gmail.com; 2 Tver State Technical University, 170026 Tver, Russia; tanovi.69@mail.ru (T.N.); victoriapetrop@gmail.com (V.P.); sulman@online.tver.ru (M.S.); 3 Polytechnic Institute, Far Eastern Federal University, 690922 Vladivostok, Russia; 4 Department of Civil Engineering, College of Engineering, Prince Sattam Bin Abdulaziz University, Alkharj 11942, Saudi Arabia; mugahed_amran@hotmail.com; 5 Department of Civil Engineering, Faculty of Engineering and IT, Amran University, Amran 9677, Yemen

**Keywords:** gypsum, recycling, energy, microcalcite, faience

## Abstract

Increasing the efficiency of using gypsum binders can be carried out by using not natural gypsum raw materials, but calcium sulfate-containing waste from various industries (phosphogypsum, borogypsum, citrogypsum, etc.). As the main source material in the work, we used gypsum-containing waste from a faience factory in the form of waste molds for casting dishes, souvenirs and plumbing fixtures. It has been established that the optimal binding system is formed by mixing powders of dihydrate technogenic gypsum from a coarse and fine earthenware factory with average particle diameters of 3.473 microns and 3.065 microns in a percentage ratio of 30:70, respectively. Using a computer software developed by the authors, which makes it possible to simulate the microstructure of a raw mixture taking into account the contact interaction of particles and calculate the average coordination number, models of binary packing of particles were constructed at various ratios of their diameters. Studies of the strength of composites obtained on the basis of bidisperse systems have shown the presence of an extremum in the region of mixtures containing 30% coarse powder. With optimal packing, a large number of phase contacts are formed due to the regulation of the grain composition of the bidisperse system. It was revealed that a brick based on the waste of two-water gypsum from earthenware production has 2.5–5 times better characteristics of compressive strength than traditional building wall products based on natural gypsum. At the same time, the strength immediately after molding is more than 3 times higher than that of traditional gypsum products. Even higher indicators are achieved when adding microcalcite in addition to the waste of earthenware production, in this case, the compressive strength is 3–6 times higher, and the strength immediately after molding is almost 3 times higher than that of traditional gypsum products.

## 1. Introduction

Traditional methods of producing gypsum building materials provide for the burning of raw materials to obtain a binder, and then the molding of products based on it and their subsequent drying [1,2,3]. It is a known method for producing gypsum products by non-firing technology [4,5]. The use of non-fired gypsum binders for the production of building products significantly increases their efficiency by eliminating the operations of obtaining a gypsum binder and drying finished products and using the technology of semi-dry pressing [6,7,8].

Regions that do not have their own stocks of gypsum raw materials are forced to incur additional transportation costs [9,10]. It is necessary to increase the efficiency of using gypsum binders in such regions by using not natural gypsum raw materials, but gypsum-containing waste from various industries (phosphogypsum, borogypsum, citrogypsum, etc.) [11,12,13]. This solves not only the issue of obtaining a cheap effective building material but also reduces the cost of exploring new gypsum deposits, extracting and transporting them, which allows you to protect the environment from pollution and dispose of accumulated waste [14,15,16].

The most promising in this case is the waste of ceramic plants in the form of used gypsum molds and models for casting [17,18]. The non-fired gypsum binder obtained on their basis makes it possible to obtain products with high physical and mechanical characteristics [19,20]. It is most effective to combine a mineral additive with lime in a multicomponent clinker-free waterproof gypsum binder and in lightweight concrete based on it [21,22].

At present, along with the hydration hardening of gypsum binders based on hemihydrate gypsum, the theory of nonhydration hardening is being developed, when the formation of crystallization structures in the “dihydrate gypsum-water” system occurs without introducing a structure-forming hemihydrate additive [23]. The process of formation of the primary crystallization structure, in this case, is due to the artificial approach of particles at the distance of intermolecular interaction, at which nuclei of accretion between large dihydrate particles (large phase) appear due to the dissolution of the smallest particles (thin phase).

According to the mechanism of structure formation in systems based on dihydrate technogenic gypsum, the formation of crystallization contacts can occur due to the achievement of supersaturation as a result of the overlap of the surface layers of dihydrate gypsum particles of different sizes, which underlies the formation of the primary structure [24]. The structure formation of systems based on gypsum dihydrate during non-hydration hardening is somewhat different from the above-described hardening schemes: there are no stages of hydration and the formation of a coagulation structure. The process, as in the case of hydration hardening, includes the stage of dissolution, however, dissolution of calcium sulfate dihydrate occurs, the solubility of which is significantly lower than the solubility of the hemihydrate. Subsequently, the condensation of the dissolved dihydrate occurs in the active centers of crystallization, the formation of a crystallization structure and the growth of crystals of the dihydrate. In the case of a non-hydration hardening scheme, the processes of structure formation with a low degree of supersaturation proceed for a long period [25].

**Figure 1:**
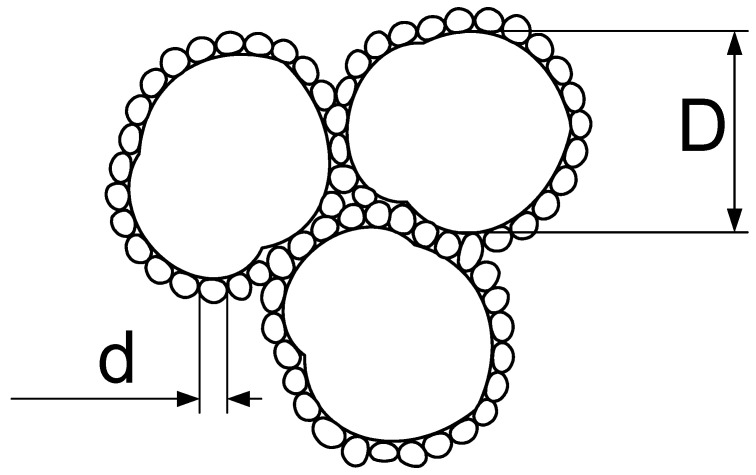
Optimal structure of non-hydration hardening systems based on a binary mixture of quasi-basal type, formed by mixing two monofractions of dihydrate gypsum.

It should be noted that not all contacts between the gypsum dihydrate particles form a crystalline structure, i.e., not all contacts are “effective”. The number of “effective” contacts, which are secondary active crystallization centers, is determined by the ratio of the sizes of the converging particles and the quantitative content of particles of different sizes in the mixture. The strength of the structure changes according to the solubility of the calcium sulfate dihydrate mixture, which is determined by the number of “effective” contacts. Hence, it follows that it is necessary to use binary mixtures of a certain type, allowing to obtain the maximum number of contacts of large and small particles in the package. Such mixtures are obtained from two “monofractions” (powder with a narrow range of particle sizes included in its composition), significantly different in dispersion, and consisting of grains with a shape close to isometric [26]. The optimal structure from the point of view of the number of “effective” contacts is formed under the condition of the presence of one grain with a small diameter (d) between grains with a large size (D) (Figure 1).

The mechanism of hardening according to a non-hydration scheme, proposed by Kuntze [8], is based on the accretion of particles of a coarse fraction of a dihydrate in dispersed systems due to the complete dissolution of particles of a fine fraction and the growth of large particles until contacts between them are formed. As noted in the work of Belov [27], for this, it is necessary to create a high supersaturation in the system and maintain it for a long time. Accordingly, the process of formation of the primary crystallization structure, in this case, is due to the artificial approach of particles at the distance of intermolecular interaction, at which nuclei of accretion between large particles of the dihydrate (large phase) appear.

The formation of a structure of this type has not been sufficiently studied. Thus, the purpose of the article is to control the formation of the structure of a gypsum composite from technogenic raw materials during non-hydration hardening. In the course of achieving the goal, the following tasks were solved:

- the use of computer modeling tools for designing the structure of a composite material, taking into account the chaotic nature of the distribution of structural elements in the volume of the material.
- finding the packing density of elements, which is necessary to determine the physical, mechanical, rheological and other properties.
- development and research of gypsum wall products based on faience production waste.

## 2. Materials and Methods

### 2.1. Materials

As the main source material in the work, we used the large-tonnage gypsum-containing waste of the Konakovsky faience plant in the form of waste molds for casting dishes, souvenirs, and plumbing fixtures. Casting forms of the faience Konakovsky plant are made from the binder Peshelansky gypsum plant, which has the best quality raw materials, which is confirmed by the data of the chemical composition of the binder obtained on its basis (Table 1). For its manufacture, a high-quality gypsum paste of the 1st grade is used, which, according to the requirements of the Russian standard GOST 4013-82, contains at least 95% calcium sulfate dihydrate, which makes it possible to classify the waste forms obtained in the production process as high-quality secondary material resources.

**Figure 2:**
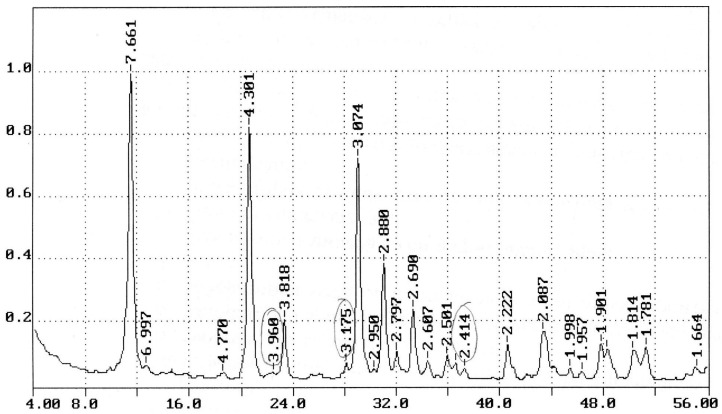
X-ray diffraction pattern of two-water technogenic gypsum, which is a waste of casting molds of a faience factory.

The genesis of gypsum-containing waste affects the structure of raw materials, and later on the properties of materials and products obtained on its basis. Waste from earthenware and ceramic production has a coarse-crystalline structure with more perfect crystals of dihydrate technogenic gypsum in comparison with waste from other industries because when using molding gypsum and the casting method for producing products (in this case, casting molds), favorable conditions are created for the recrystallization of dihydrate gypsum, the consolidation and enlargement of crystals in the free pore space. The X-ray diffraction pattern of the Konakovskiy man-made gypsum paste is shown in Figure 2.

The provided X-ray diffraction pattern is characteristic of gypsum dihydrate (diffraction lines 7.661; 4.301; 3.818; 3.074; 2.885; 2.797; 2.69; 2.603; 2.501; 2.222; 2.144; 2.083; 1.998; 1.903; 1.81; 1.67 Ǻ). Diffraction lines 3.50; 1.852 A indicate a low content of anhydrite (CaSO_4_). Impurities present in an insignificant amount in the studied gypsum sample are presented in the form of feldspars—Ca {Al_2_Si_2_O_8_} (diffraction lines 3.187; 2.51; 2.135; 1.834 A) and pyrite—FeS_2_ (lines 2.696; 2.411; 2.21 A). Diffraction reflections of calcite (CaCO_3_), quartz (SiO_2_), and clay minerals were not found in the X-ray diffraction pattern.

**Figure 3:**
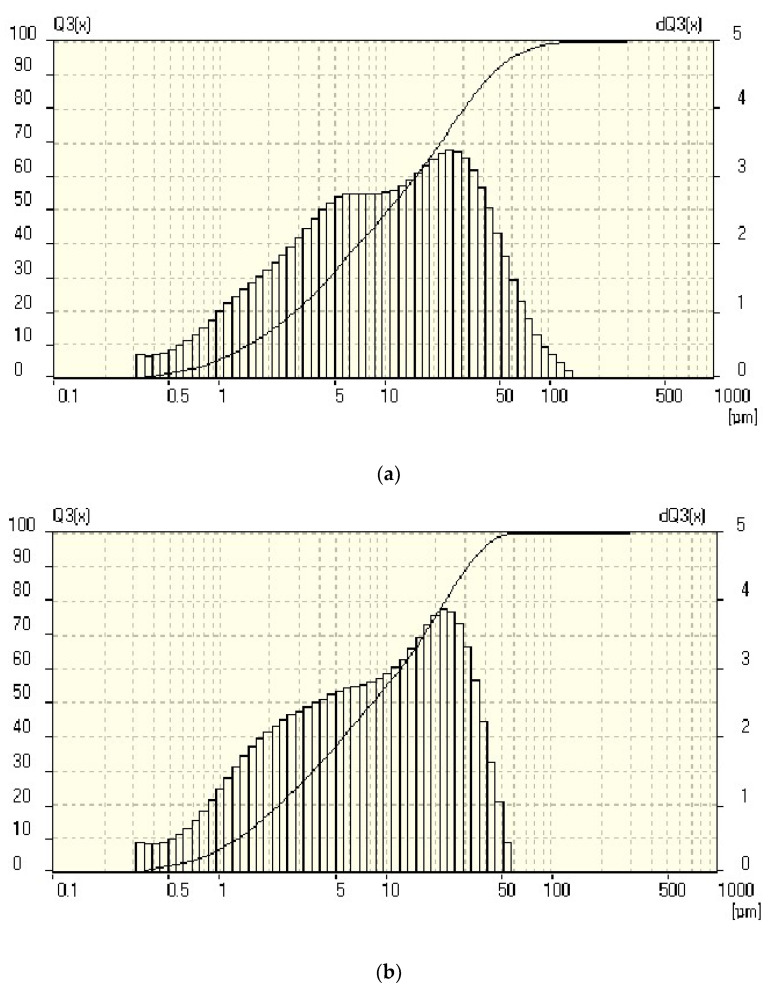
Granulometric compositions of finely dispersed gypsum powders obtained based on wastes of a faience factory: (**a**) d_av_ = 3.884 μm; (**b**) d_av_ = 3.296 μm.

The data on the granulometric composition of the finely dispersed gypsum powders were obtained using a diffraction laser analyzer (Figure 3).

The histogram of the distribution of particles in the composition of the powder, in accordance with Figure 3a, corresponds to the normal distribution law, the top is displaced relative to the center towards larger particles. The average particle size in the composition of the raw mix is 3.884 μm. The integral curve is flat—the range of particle size scatter, according to the histogram, is rather narrow—from 2 to 100 μm.

The histogram of the distribution of particles in the composition of the powder, in accordance with Figure 3b, corresponds to the normal distribution law, the top is displaced relative to the center towards larger particles. The average particle size in the composition of the raw mix is 3.296 μm. The range of particle size scatter, according to the histogram, is rather narrow—from 2 to 50 μm.

As a modifying additive, we used a complex additive in the composition of: microcalcite, Portland cement with slag, and a superplasticizer. For this, wastes of ground marble were used (microcalcite with a content of crystalline calcium carbonate CaCO_3_—97%) (White marble, Chelyabinsk, Russia). The average particle size in the composition of the microcalcite powder is 5 μm, the specific surface of the powder is 2100–2200 m^2^/kg. We also used Portland cement with slag CEM III 32.5 produced by Magnitogorsk Cement and Refractory Plant. Melflux 5581 F (BASF, Ludwigshafen am Rhein, Germany) was used as a polycarboxylate superplasticizer (SP). In addition, ammonium alum was used as a modifying additive in the work.

### 2.2. Mix Design

Table 2 shows five developed samples: the first is completely gypsum from faience production waste, in the second cement and ammonium aluminate are added, in the third 10% microcalcite is added to the faience production waste, in the fourth there is the superplasticizer, in the fifth all the listed components are present excluding ammonium aluminate.

### 2.3. Methods

#### 2.3.1. Raw Material Properties

Due to the fact that the two-water technogenic gypsum is a lumpy material, calcium sulfate dihydrate powders were obtained by grinding in a jaw crusher before passing through a No. 5 sieve and subsequent grinding in a laboratory ball mill.

For the particle size analysis of gypsum powders of various fineness of grinding, waste of rock crushing, laser analysis was used in the study using an Analysette 22 diffraction particle analyzer (Fritsch, Idar-Oberstein, Germany).

The specific surface of the powders was determined on a PSH-11SP device (Pribory Khodakova, Moscow, Russia) by the air permeability method, based on the measurement of the hydraulic resistance that a layer of compacted powder has to air sucked through it.

The phase and chemical composition of gypsum-containing waste powders was determined by differential thermal, X-ray structural and chemical analysis.

The solubility of the raw mixtures was determined by the value of the electrical conductivity of the solutions using a Multitest KSL-101 conductometer (Meratest, Moscow, Russia).

In view of the fact that one of the factors determining the formation of the internal structure of a dispersed system is the bulk density, which determines the formation of the maximum number of primary contacts between the particles of gypsum dihydrate, and the number of contacts is determined not only by pressure, but also by the grain size composition. In this regard, the work investigated the bulk density of powdery raw mixtures of various grain size compositions. The microstructure of calcium sulfate dihydrate powders and their mixtures was assessed by electron microscopy using an MBS-1 stereoscopic microscope, and the microstructure of a gypsum stone using a Scan 4 scanning microscope.

#### 2.3.2. Strength Properties of Concrete

During molding, the method of semi-dry pressing on a laboratory hydraulic press was used. The gypsum specimens were hardened at normal temperature in a desiccator at an ambient humidity of more than 95%. Strength characteristics were determined on cylinder specimens 25 mm × 25 mm in size in accordance with standard test methods. Pressing equipment was used: a 10-ton laboratory press with a measurement range of 0–100 kN (graduation 1 kN), as well as a manual hydraulic press PRG-1-50 (Tekhmash, Neftekamsk, Russia). The sample was centered on the base plate of the press. The average rate of increase in the load during the test was (1.0 ± 0.5) MPa/s. The compressive strength of an individual specimen was calculated as the quotient of the breaking load divided by the specimen area. The compressive strength for the composition was calculated as the arithmetic mean of the test results of the samples.

## 3. Results and Discussion

### 3.1. Selection of the Optimal Grain Composition

**Figure 4:**
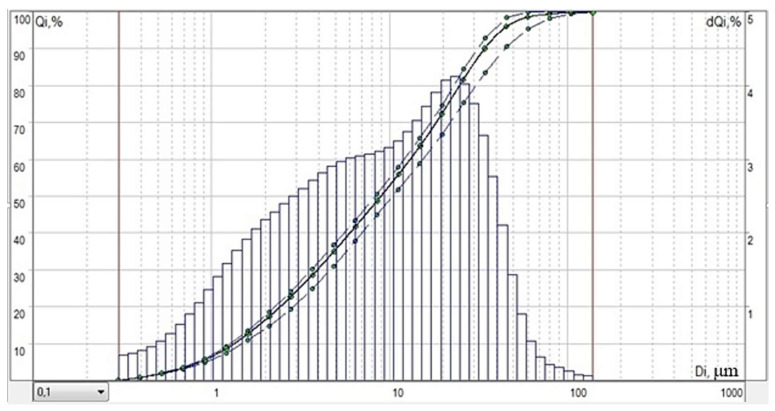
Particle size of a bidisperse mixture of optimal grain size composition based on coarse and fine gypsum powders obtained from the waste of a faience factory.

A bidisperse system based on coarse and fine gypsum powders obtained based on technogenic waste from a faience factory was investigated. The rational binder system is formed by mixing powders of dihydrate technogenic gypsum from a faience factory of coarse and fine grinding with average particle diameters of 3.473 μm and 3.065 μm in a percentage ratio of 30:70, respectively. The rationalized system is more active due to the formation of a significant number of gaps and slots with a negative curvature of the surface, which contributes to an increase in its solubility and the efficiency of the structure formation process (an increase in the formation rate and an increase in the contact area). Figure 4 shows the granulometric composition of the bidisperse mixture with the most dense packing of particles and the smallest number of contacts.

The histograms of the distribution of particles in the composition of powders of two-water technogenic gypsum from a faience factory of coarse and fine grinding with an average particle diameter of 3.884 μm and 3.296 μm, respectively, correspond to the normal law, the tops of the distribution curves with an increase in dispersion shift towards small particles. With prolonged grinding, the specific surface of the powders decreases due to the intensifying process of aggregation.

Grain composition plays an important role in the process of structure formation and subsequent strength gain of gypsum systems. The presence of a fine fraction with the highest specific surface area is invaluable, but reaching a specific surface area of the order of 900–1000 m^2^/kg requires expensive equipment and complication of technology due to the high adhesion of gypsum particles. Therefore, a larger fraction is required.

The task of this work was solved by selecting the optimal granulometric composition of a bidispersed system based on gypsum powders of coarse and fine grinding, subject to the formation of the maximum number of contacts, the mechanism of non-hydration hardening, so a small particle should be located in the interval between two coarse particles.

**Figure 5:**
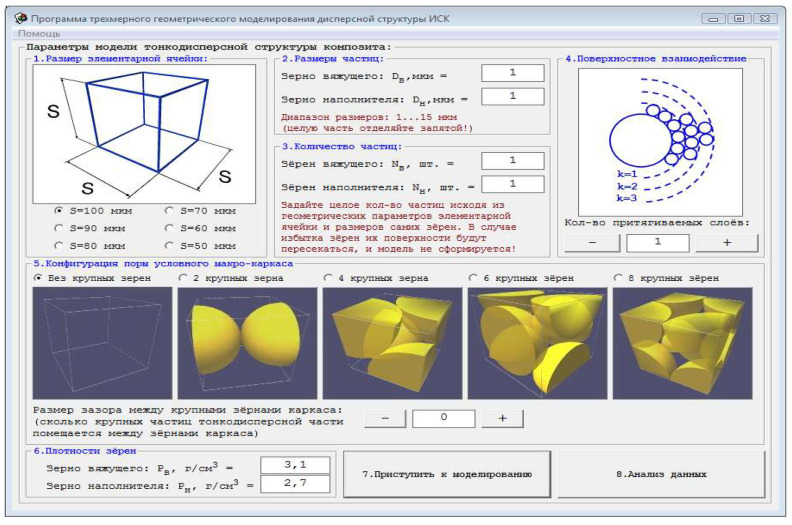
Computer software that allows you to simulate the microstructure of the raw mix.

With the use of a computer program developed by the authors (Figure 5), which allows simulating the microstructure of a raw mixture taking into account the contact interaction of particles and calculating the average coordination number, models of binary packings of particles were constructed at various ratios of their diameters.

The algorithm of the program allows you to study the process of packing a bifraction array of particles, with the possibility of setting their sizes and numbers, which makes it possible to obtain various structures of binary systems. The calculation of the quantitative characteristics of the model is carried out according to stereometry formulas by summing the volumes of spherical bodies. In the course of the work program of the program, a three-dimensional model of particle packing is analyzed; the calculated volume of filled particles; pore volume; the number of contacts formed on the surface of a large particle; the number of contacts of small particles on a single surface of a large particle.

**Figure 6:**
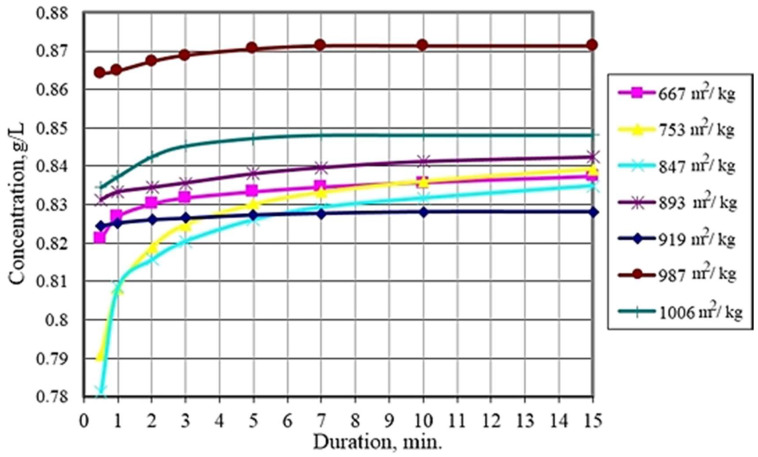
Saturation kinetics of solutions of calcium sulfate dihydrate powders of different dispersion.

The method of summing static collisions of elements is used to calculate the actual number of contacts of particles at a certain degree of filling the elementary volume with them. As an object of modeling, a mixture of powders obtained based on two monofractions was chosen: a large one with a particle size (diameter) D1 and a small one with a particle size (diameter) D2. The crystalline structure of the non-hydration hardened dihydrate technogenic gypsum is most consistent with cubic packing. Based on the above positions, a face-centered cubic packing of large particles was laid on the basis of the spatial computer model. With the help of this program, setting the initial parameters, it is possible to obtain a quantitative estimate of the packed array of spherical particles (Figure 6).

In the computer model, the packing of small particles is carried out according to the algorithm of rolling particles (“drop and roll”).

The optimal structure from the point of view of the number of “effective” contacts is formed under the condition of the presence of one grain with a small diameter d between grains with large sizes D. The ratio of sizes (diameters) of elementary particles in a binary system ranges from 1 to 16. With an increase in the difference in particle sizes the proportion of large particles, which have larger coordination numbers than in systems with the same particle size, increases. An increase in the total coordination number of particles in the system, which characterizes the number of phase contacts, should lead, in particular, to an increase in the strength of structures obtained based on binary mixtures of normalized composition.

The results of a computational experiment to optimize the internal structure using the obtained model show that the optimal content of coarse powder is 30–40%.

### 3.2. Influence of Optimal Packaging on the Physical and Mechanical Properties of the Composite

The solubility of disperse systems of various degrees and character of dispersion of monofraction powders and their bifraction mixtures based on calcium sulfate dihydrate of the faience plant was investigated, which was determined by the value of the electrical conductivity of solutions using a conductometer “Multitest KSL-101” (Figure 6).

**Figure 7:**
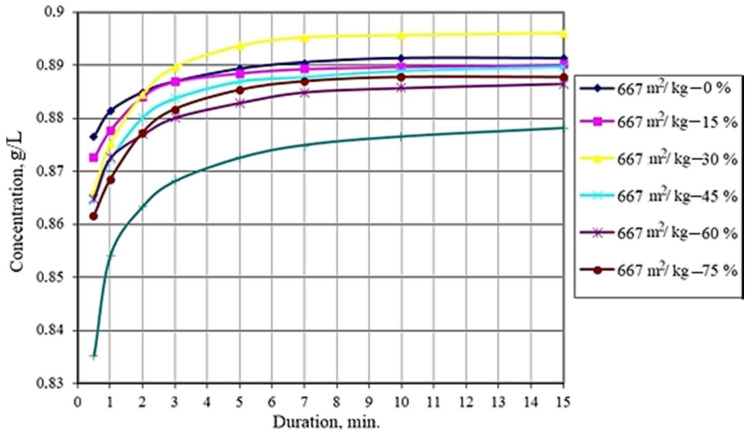
Saturation kinetics of solutions of bidisperse mixtures of calcium sulfate dihydrate powders with different contents of coarser powder.

The studies carried out show that the solubility of dispersed systems of dihydrate gypsum of technogenic genesis depends on the fineness of grinding. With an increase in the specific surface of the powders from 667 to 987 m^2^/kg, the solubility increases. With a further increase in the specific surface area to 1006 m^2^/kg, the solubility decreases, which is due to a decrease in the number of defects on the particle surface. A mixture containing 30% powder with a specific surface area of 667 m^2^/kg (Figure 7) possesses the highest solubility among bidispersed mixtures. Both individual powders and their mixtures are characterized by the same dissolution rate and solution saturation time under given conditions.

Thus, the results of experiments on the study of the solubility of monofraction powders and their bifraction mixtures based on industrial waste show that the solubility depends on the particle size for both powders and their mixtures, increasing with increasing dispersion.

The optimal ratio of powders of different fineness established by the model is confirmed by studies on the solubility of bidisperse systems. The maximum value is reached when the content of coarse powder in the binary mixture is 30%.

**Figure 8:**
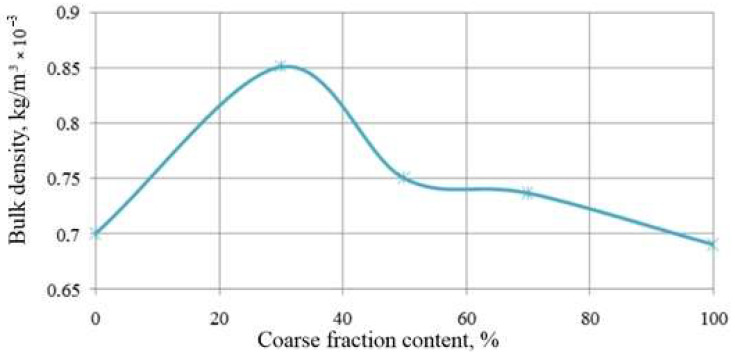
Dependence of the bulk density on the content of coarse calcium sulfate dihydrate powder based on industrial waste with specific surfaces of 667 and 1006 m^2^/kg.

The bulk density of loosely packed bidisperse mixtures of technogenic genesis depends on the percentage of individual powders of calcium sulfate dihydrate (Figure 8). The maximum density is typical for mixtures with a coarser powder content of 30%, which is comparable to the previously obtained results in terms of solubility. The results obtained are also consistent with the developed mathematical model.

In order to study the physical and mechanical characteristics, samples were molded by the method of semi-dry pressing on a laboratory hydraulic press. Investigations of pressed materials based on dihydrate technogenic gypsum were carried out on samples-cylinders with a size of 25 mm × 25 mm. Solid gypsum specimens at normal temperature in a desiccator in an environment of more than 95%.

**Figure 9:**
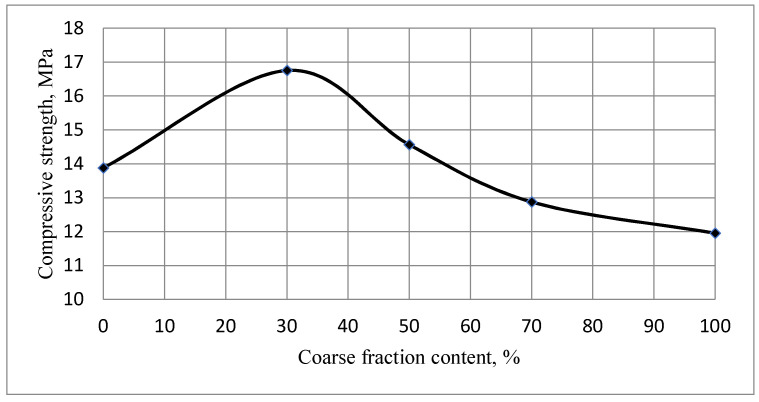
Dependence of the strength of the gypsum composite on the content of the coarse fraction of a mix of powders of dihydrate gypsum of technogenic genesis with S_sp_ = 667 m^2^/kg and S_sp_ = 1006 m^2^/kg on the 7th day of hardening at W/S = 0.13.

Studies of the strength of composites obtained based on bidisperse systems have shown the presence of an extremum in the region of mixtures with a content of 30% coarse powder (Figure 9). With optimal packing, it forms the largest number of phase contacts due to the regulation of the grain size composition of the bidisperse system.

Due to this, the obtained experimental data on the solubility, bulk density of single-fraction gypsum powders and bifraction mixtures based on them, as well as the strength of gypsum composites coincide with the calculated ones, which confirms the efficiency of the proposed mathematical model. According to the research results, the optimal content of coarse powder is 30%, using the model, the optimal ratio is obtained—30–40%.

**Figure 10:**
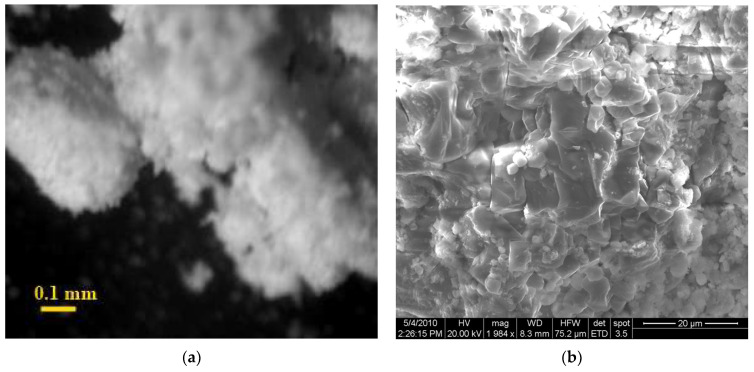
Microstructure: (**a**) powder of dihydrate technogenic gypsum; (**b**) composite based on two-water technogenic gypsum, obtained on the basis of a binary mixture of normalized grain size composition.

Figure 10a,b show the internal microstructure of a powder of two-water gypsum of technogenic genesis, as well as a composite of a binary mixture of normalized grain size composition.

**Figure 11:**
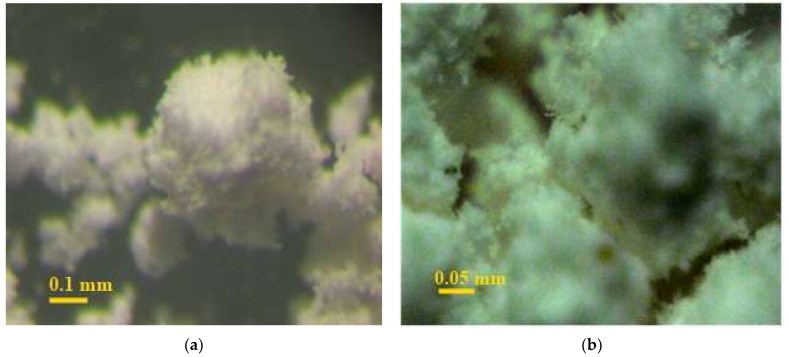
Internal microstructure of a binary dispersed system based on powders of calcium sulfate dihydrate with magnification: (**a**) ×84, (**b**) ×200.

The structure of binary mixes of dihydrate powders is characterized by the retention of coarse conglomerates and an increased content of highly dispersed particles. This structure of the powder creates the prerequisites for the formation of the maximum number of phase contacts. Cohesive interaction between large and small particles determines the formation of cluster-type conglomerates in the system. These conglomerates are characterized by different sizes, which are determined by the size of the cluster-forming particle (Figure 11a). The formed clusters, in turn, form a fractal structure, which is confirmed by microscopic studies, the microstructure of fine conglomerates (Figure 11b) is similar to the general structure.

The microstructure consists of large conglomerates (clusters), in which the inter-conglomerate space is filled with dispersed particles of different sizes. This increases the density of the entire system.

The conglomerates are large particles surrounded by a finely dispersed phase, which contributes to the creation of an optimal structure of the non-hydration hardened material. The grains forming the granules are quite different in size: the mixture contains both sufficiently large particles and highly dispersed particles. The contact of large grains with each other occurs only through a layer of small particles. Very small parts of the grains of the finest fractions form conglomerates of monodisperse composition.

Thereby, studies of the grain size composition of dispersed systems based on two-water technogenic gypsum, composed of polydisperse powders in various combinations, confirm the presence of conglomerates of various sizes in the system, as well as particles of different sizes that meet the requirements of non-hydration hardening. Optimal from the point of view of the theory of non-hydration hardening is a dispersed system composed of powders of dihydrate gypsum of natural and technogenic genesis with a content of coarse and fine powders in a ratio of 30:70, respectively, having an optimal ratio of “maximum peaks” in the composition of the dispersed system, satisfying the theoretical provisions of the theory non-hydration hardening.

Thus, optimization of the granulometric composition of raw mixtures based on dihydrate gypsum of technogenic genesis makes it possible to increase the strength of the resulting composite by increasing the phase contacts in the hardening system.

The regulation of the grain size composition and the increase in the dispersion of dihydrate technogenic gypsum are the main factors in obtaining high-strength gypsum paste, high quality materials and products based on it.

The economic effect in the production of non-fired gypsum pressed bricks is ensured by reducing energy consumption for mining, transportation, grinding and firing of raw materials, as well as for drying finished products.

### 3.3. Physical and Mechanical Characteristics of Gypsum Building Products

The physical and mechanical properties of a gypsum composite based on gypsum dihydrate modified with microcalcite are given in Table 3.

The results obtained make it possible to use this composite for the production of wall building products. Comparative characteristics of the properties of gypsum non-fired bricks on various binders are given in Table 4.

It was revealed that a brick based on waste of dihydrate gypsum from earthenware production has 2.5–5 times better characteristics of compressive strength than traditional building wall products based on natural gypsum. At the same time, the strength immediately after molding is more than 3 times higher than that of traditional gypsum products. Even higher rates are achieved when adding microcalcite in addition to the waste of earthenware production, in this case, the compressive strength is 3–6 times higher, and the strength immediately after molding is almost 3 times higher than that of traditional gypsum products.

The economic effect in the production of non-fired gypsum pressed bricks is ensured by reducing energy consumption for mining [28], transportation, grinding and firing of raw materials, as well as for drying finished products.

## 4. Conclusions

Increasing the efficiency of using gypsum binders can be carried out by using not natural gypsum raw materials, but gypsum-containing waste from various industries (phosphogypsum, borogypsum, citrogypsum, etc.). As the main source material in the work, we used gypsum-containing waste from a faience factory in the form of waste molds for casting dishes, souvenirs and plumbing fixtures. It was developed five samples: the first is completely gypsum from faience production waste, in the second cement and ammonium aluminate are added, in the third 10% microcalcite is added to the faience production waste, in the fourth there is the superplasticizer, in the fifth all the listed components are present excluding ammonium aluminate.

The use of computer modeling tools for designing the structure of a composite material, taking into account the chaotic nature of the distribution of structural elements in the volume of the material. The following provisions of scientific novelty have been obtained:

- In gypsum powders, the formation of fractals at different levels of dispersion is possible due to the adhesion of small particles to large ones. An important point here is the similarity of material structures at scale levels, the so-called fractal nature of the structure, which in turn fragments the optimization problem and expands the range of structure optimality parameters.
- Formation based on a bidispersed system of non-hydration hardening of aggregates or clusters with stirring and moistening simplifies the pressing process. The rational binder system is formed by mixing powders of dihydrate technogenic gypsum from a faience factory of coarse and fine grinding with average particle diameters of 3.473 μm and 3.065 μm in a percentage ratio of 30:70, respectively.
- Optimization of the system as a result of the formation of a significant number of gaps and slots with a negative curvature of the surface contributes to an increase in its solubility and strength, the efficiency of processes during non-hydration hardening of gypsum dispersed systems. The studies carried out show that the solubility of dispersed systems of dihydrate gypsum of technogenic genesis depends on the fineness of grinding. With an increase in the specific surface of the powders from 667 to 987 m^2^/kg, the solubility increases. With a further increase in the specific surface area to 1006 m^2^/kg, the solubility decreases, which is due to a decrease in the number of defects on the particle surface
- The most decisive factor in the formation of a dispersed structure is the ordering of particles, that is, the packing of material grains at all scale levels, the most important of which is the morphology of particles (size, shape, etc.).
- It was revealed that a brick based on waste of dihydrate gypsum from earthenware production has 2.5–5 times better characteristics of compressive strength than traditional building wall products based on natural gypsum. At the same time, the strength immediately after molding is more than 3 times higher than that of traditional gypsum products. Even higher rates are achieved when adding microcalcite in addition to the waste of earthenware production, in this case, the compressive strength is 3–6 times higher, and the strength immediately after molding is almost 3 times higher than that of traditional gypsum products.

## Figures and Tables

**Table 1:** The chemical composition of the gypsum binder β—modification of the Peshelansky gypsum plant.

SiO_2_	Al_2_O_3_	TiO_2_	Fe_2_O_3_	CaO	MgO	SO_3_	Na_2_O	K_2_O	P_2_O_5_	F
0.8	traces	traces	–	37.52	0.10	53.78	0.05	0.007	–	–

**Table 2:** Mix design.

Mix ID	Description	Mixture Composition,% (by Dry Matter)					Water–Solid Ratio
		Dihydrate Gypsum (25% Coarse: 75% Fine)	CEM III 32.5	Micro-Calcite	SP	Ammonium Alum	
1	Brick based on waste of dihydrate gypsum from faience production	100	-	-	-	-	0.06
2	Brick based on wastes of dihydrate gypsum from faience production with ammonium alum	71	28.5	-	-	0.5	0.28
3	Brick based on wastes of dihydrate gypsum from faience production with the microcalcite	90	-	10	-	-	0.12
4	Brick based on wastes of dihydrate gypsum from faience production with CEM III 32.5 and SP	86	9	-	5	-	0.1
5	Brick based on wastes of dihydrate gypsum from faience production with the microcalcite, CEM III 32.5 and SP	76	9	10	5	-	0.08

**Table 3:** Physical and mechanical properties of a gypsum composite based on gypsum dihydrate modified with microcalcite (28 days of hardening).

Properties	Units of Measurement	Values
Average density	kg/m^3^	1900
Flexural strength	MPa	3.83
Compressive strength	Mpa	68.0
Water resistance	-	0.6

**Table 4:** Comparative characteristics of the properties of gypsum non-fired bricks on various binders.

Properties	1	2	3	4	5
Average density, kg/m^3^	1960	1650	1140	1050	1520
Compressive strength, MPa	25	10.5	5.3	10.0	36
Compressive strength immediately after pressing, MPa	3.6	1.1	1.1	0.9	5.0

## Data Availability

Not applicable.
